# Improving the Timeliness of Discharge Summary Communication: A Quality Improvement Project

**DOI:** 10.7759/cureus.32602

**Published:** 2022-12-16

**Authors:** Luke Glover, Georgia Wright, Simon Brackley, Miles Edwards, Lauren Eddy

**Affiliations:** 1 Medicine and Surgery, Royal Devon University Healthcare NHS Foundation Trust, Exeter, GBR

**Keywords:** delayed communication, hospital discharge, primary care, hospital summary, communication, discharge summary

## Abstract

Introduction

A recognised problem in the Royal Devon & Exeter Hospital and across the NHS is that discharge summaries are often sent to general practitioners (GP) long after the patient is discharged, or not at all. This is a safety issue when for example the summary includes time-sensitive requests for the GP or information relevant to ongoing care.

Methods

A quality improvement project was devised to tackle this important problem. First, the Royal Devon & Exeter Hospital's electronic patient record system was used to construct a report allowing measurement of the scale of this problem and stratification by factors such as discharging department or ward. This report identified that 22.6% (12,965/57,367) of discharge summaries are sent outside the Trust’s target (two working days from discharge). A three-pronged approach was devised targeting discharges of deceased patients using educational material, discharges from a medical ward using an automated list, and finally optimising the technical steps required to send a discharge summary to attempt to reduce the delay.

Results

Plan, do, study and act (PDSA) cycles were implemented, one targeting discharges for deceased patients and another targeting discharges from a medical ward. Though not sustained, the former resulted in a six-week increase in the percentage of discharge summaries sent within the target from 50% to 80%. The latter did not lead to improvement due to a number of factors including workload in the midst of a global pandemic and other factors explored in a root cause analysis.

The most ambitious intervention aimed to automate an administrative step, which proved challenging due to software and human factors. As such this intervention was not completed during the study period.

Conclusion

The sending of discharge summaries is often delayed and this has potential consequences for patient care. This study has used the hospital's electronic patient record system to create a report which provides detailed information on areas with the most potential for improvement. Though the targeted interventions were respectively nonsustained, unsuccessful and not implemented, this data can suggest reasons behind poor performance and therefore targets for future interventions, illustrating great sustainability.

## Introduction

Discharge summaries are documents written by a patient’s hospital doctor which aim to summarise their admission, including key events, test results, medication changes and actions for follow-up. Each patient should have a discharge summary signed and sent to their general practitioner (GP) when leaving the hospital; for a number of reasons this often does not happen. Discharge summaries are vital for ongoing patient care and frequently contain important tasks for GPs [1] such as arranging blood tests, requesting clinical reviews, or restarting vital medication. Delayed sending therefore not only creates additional workload and dissatisfaction for GPs [2,3], hospital doctors and other members of the multidisciplinary team including pharmacists [4], but also risks patient safety [5-7]. Various methods have been trialled in the past to improve communication between primary and secondary care, including direct phone conversations between hospital and primary care doctors on discharge [8] and post-hospitalisation multi-professional clinic visits [9], with little evidence for success. Clearly, this issue remains, and it is believed by this project team to be largely a result of increasing workload and clinical pressures, especially in the midst of a global pandemic [10].

The Royal Devon & Exeter Hospital (RD&E) is a large teaching hospital, discharging about 3500 patients per month. A total of 22.6% of these patients do not have discharge summaries sent to their GPs within two working days of their discharge, which is the national target [11]. This is a significant number and could lead to adverse events, especially as medication changes are often enacted [12] as well as complex plans for follow-up investigations. Indeed one study found that on discharge nearly half of patients in their small cohort experienced at least one medical error due to poor communication between secondary and primary care [13].

The project team consisted of junior doctors working within the trust, each of whom was acutely aware of the pressure from senior management and from GPs to improve this issue, not least because several complaints levied at the trust relate to unsent or delayed discharge summaries. Rather than simply addressing the ever-growing backlog of unsent summaries, the team was eager to address the source of the problem. This quality improvement project aimed, over the course of nine months, to increase the proportion of discharge summaries sent within two days to 90% across all wards and departments, by July 2022. A few weeks into the project and after consultation with colleagues, this timescale target was refined from two days to two working days to reflect national targets and the (usually) five-day-work-week pattern of primary care work.

## Materials and methods

Data collection

The first step of this project was ensuring the collection of reliable, continuous data. Since 10th October 2020, the RD&E has used an electronic patient record system (Epic). Epic is an electronic health records system for hospitals and large practices. It includes features such as medical templates, blood results and prescriptions. It also incorporates ways for patients to easily access their own health records, facilitating greater involvement in their own healthcare. Worldwide, more than 250 million patients have an electronic health record in Epic [14]. This powerful and extensive database allows users to create customised ‘reports’ that mine specified data and can prove a useful tool for quality improvement [15].

The initial report run from 10th October 2020 to 3rd March 2022 identified active discharge “deficiencies” within Epic; that is, it produced a list of patients who have been discharged with no documented summary sent from that admission. It soon became evident that this data does not include patients whose discharge summary was significantly delayed but eventually sent (so there is no longer an active deficiency). To solve this, a second report was created that pooled all discharges at the RD&E during the same time period and also included the date of discharge and date of discharge summary communication. Exporting this to database software and using simple code then allowed the identification of discharges where the length of time between discharge and resolution of the discharge deficiency was greater than two working days. In this way, the percentage of discharge summaries which are not sent within two working days could be calculated.

This amalgamated data could also be stratified based on the categorised data included in the report: ward, department, specialty, day of week, time of day and mode of discharge. In this way, the areas with the most potential for improvement were identified and could be targeted.

**Figure 1:**
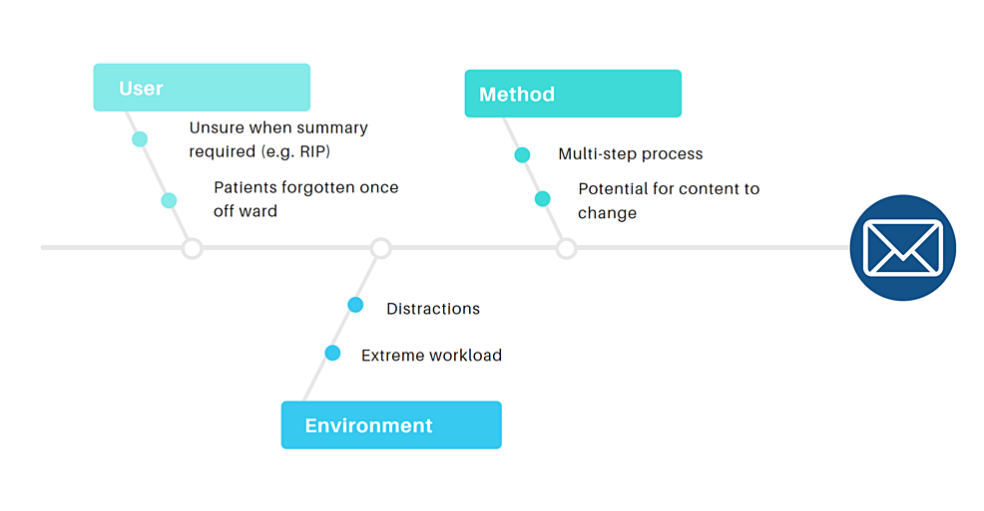
Root cause analysis This root cause analysis shows potential factors, grouped into three categories, which could lead to delayed sending of discharge summary communications to GPs. These formed part of the basis for choosing areas to target. RIP: Rest-in-peace or "discharge as deceased"; GPs: General practitioners.

A root cause analysis (Figure 1) was also performed which identified potentially valuable targets for change.

**Figure 2:**
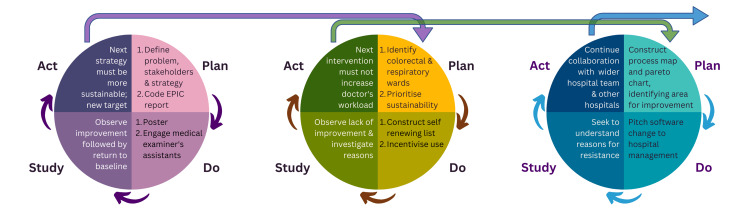
Graphical summary of the plan-do-study-act methodology used in this study This graphic summarises the approach taken by the project in targeting three different areas based on the root cause analysis and areas with potential for improvement. The first, left-most cycle targeted "RIP discharges," the middle cycle targeted "patient lists" and the right-most cycle targeted "automatic sending." RIP: Rest-in-peace or "discharge as deceased"

Because of the powerful nature of the project’s “live” data collection, easily stratified by ward, specialty, department and more, it was feasible to put in place three PDSA (plan, do, study and act) cycles independently, allowing almost simultaneous tests of change as each intervention was trialled. Three areas were targeted based on the root cause analysis and areas with potential for improvement: discharges of deceased patients, modifying patient lists and automatic sending of unsigned summaries (Figure 2).

Discharges as deceased

The first PDSA cycle involved patients who die in the hospital. These patients require a “discharge as deceased” or "Rest in peace (RIP) discharge" summary to be sent to their GP to inform them of the death and cause (or likely cause if being investigated by the coroner).

At the RD&E, junior doctors are summoned to the Medical Examiner’s (ME) Office by the Medical Examiner's assistants to complete death certificates. They are then aided in filling out the certificate by that same assistant, who will guide them through the necessary paperwork. These individuals are well aware of the importance of RIP discharge summaries, because part of their role is to inform GP practices of patient deaths, communicate with relatives, and deal with complaints and grievances. These key members of staff are well placed to remind junior doctors to write the discharge summary as part of their usual guidance on the other paperwork to be completed whilst the doctor is in the office. Therefore, the first PDSA cycle undertaken by this project was to involve the ME assistants as stakeholders, and ask them to remind doctors to fill out summaries while in the office. This was in parallel with a poster-based educational intervention encouraging doctors to stay in the quiet and calm environment of the office to complete and send the summary, rather than returning to the ward.

It was anticipated that the poster-based element of this intervention would be unsustainable, and it was intended to be a ‘jump-start’ to the more long-lasting element, envisioned as the involvement of the ME’s assistants. It was hoped that harnessing the enthusiasm of these staff members and giving them explicit license to discuss discharge summaries with doctors would create a self-motivated and therefore self-sustaining intervention.

Patient lists

The second intervention involved patient “lists.” It was inspired by the experiences and feedback of junior doctors on surgical placements, where there is an emphasis on lists and ‘clearing the list’, or handing over all the patients who were seen that day. Having as short a list as possible is seen as a marker of efficient work, and doctors are in general highly motivated to achieve this. It was hoped that by making the simple change of adding discharged patients with unsent discharge summaries to the list, this would provide extra visual reminders and motivation to complete the paperwork. In practical terms the intervention involved building a self-renewing electronic list of patients discharged from the ward in the last 30 days who did not have a discharge summary sent. This is already a core list in Epic and merely needed to be combined with each user's existing patient list with a relatively simple drag and drop.

The intention was that, compared to the first, opportunistic and education-based intervention, this would be a permanent change to workflow and therefore would be sustainable. Initially, the colorectal surgery firm was targeted, as the surgical firm with a high rate of discharges and with significant support for the project from several of the senior colorectal staff including consultants. However, due to unforeseen quirks in the data entry process in Epic, a variable proportion of patients under the care of the colorectal team may be registered as under ‘general surgery’ instead, which made test-of-change measurement unreliable. As a result, the target for this cycle was altered to the respiratory wards. The respiratory wards have a high rate of patient discharges and with high numbers of junior doctors was also a convenient target for the intervention.

The main anticipated problem with this intervention was uptake; it was uncertain whether doctors would be willing to switch to this new list. Making the change required a small alteration to the normal list set up within Epic and following this, the appropriate list needed to be made "default" so that it is the first list to appear once logged in to Epic. To mitigate against this potential confounding factor, it was crucial that adding the process was made as easy as possible; this was achieved by providing screenshots of each involved step in an introductory email. Additionally, a chocolate-based incentive for providing screenshot evidence of the implemented list was offered. The self-renewing nature of the list was intended to maximise sustainability. Once doctors knew how to add the list to their profiles, it would constantly update to show newly discharged patients without summaries.

Automatic sending

The third and most ambitious quality improvement cycle was squarely aimed at targeting what was widely perceived amongst doctors to be the main barrier to achieving timely sending of discharge summaries - the administrative burden of the process.

Drawing up a process map clarified the sequence of steps required to send a discharge summary. First, one must write a discharge summary (which can be contributed to throughout the admission by multiple authors). This written summary must be refreshed to ensure up-to-date discharge orders are included, before being signed by the final author. In this way, the doctor who has put their signature to the summary may not be the patient's regular doctor and indeed in many cases may not have even met the patient, for example when discharging patients out of hours. At this stage, the discharge summary is “signed” but is not yet “sent.” In order to take the final step, the doctor must go through the process of creating a “communication,” copying it to the patient’s GP and adding the discharge summary to the letter, before clicking “send.” Only then is the discharge process complete and the summary sent. The team looked at each stage of this process to identify areas for improvement.

From informal discussions with colleagues, some expression of dissatisfaction with the separation between “signing” and “sending” a discharge summary was gathered. The final administrative step of "sending" was not being performed either because of time pressures or because the discharge summary had been signed in advance and not sent as the patient had not physically left the hospital yet. The problem here may partially derive from the staffing shortages and rota patterns of junior doctors; the doctor who wrote the discharge summary is not guaranteed to be the same doctor seeing the patient the next day. As a result, the signed summary is never sent. In order to seek objective evidence that this was a significant deficit in the process, a Pareto chart was created to analyse the impact a change here would have. The results showed that in ideal circumstances, removing the separate step of creating a communication (i.e., automatically sending signed discharge summaries) may play a significant role in reducing the number of delayed or unsent discharge summaries.

The aim of this cycle was therefore to eliminate this last step so that on signing the discharge summary, it is automatically sent to the patient’s GP. Several issues were predicted with this change. First were the technical, financial and managerial barriers inherent in enacting a large, potentially complicated software change. University College London Hospitals NHS Foundation Trust, which also uses Epic, has implemented automatic sending of signed discharge summaries and so it was felt that this potential obstacle could be overcome.

## Results

The pre-intervention measurement using Epic reports for the period of 10th October 2020 to 3rd March 2022 identified that 22.6% (12,965/57,367) of patient discharges do not have a discharge summary sent within two working days. Discharges on Fridays and discharges of deceased patients were the poorest performing day and method of discharge respectively.

**Figure 3:**
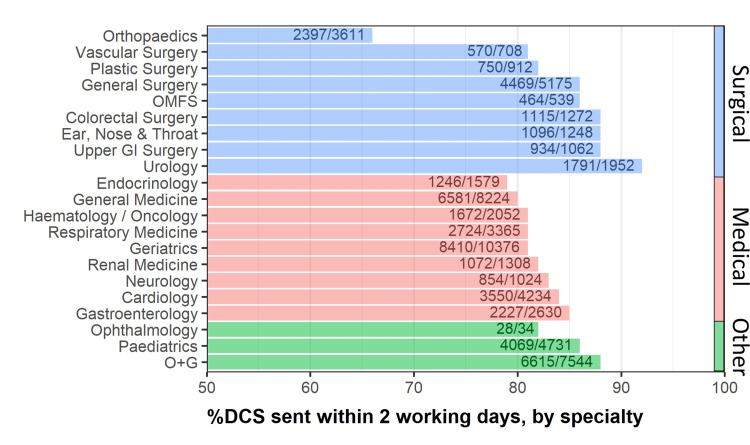
Percentage of discharge summaries sent within two working days by specialty This figure shows one example of the powerful nature of the data collected - the percentage of discharge summaries (DCS) sent within two working days could be stratified in great detail, including by hospital specialty. OMFS: Oral and Maxillofacial Surgery; O+G: Obstetrics and Gynaecology; GI: Gastrointestinal

This amalgamated data was further stratified by ward, specialty, day and time of discharge, and method of discharge (Figure 3).

The trust’s electronic patient record, combined with the powerful design of the report devised for this project, enabled continuous data collection that could be grouped by 24 hours, seven days, or any other interval. Given that the target for discharge summary sending in the trust is two working days, grouping data by seven days was felt to be appropriate for observing any changes that may follow intervention.

**Figure 4:**
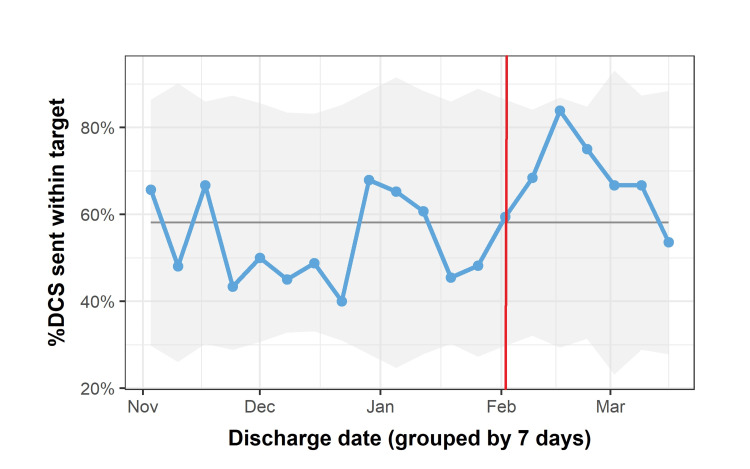
Percentage of discharge summaries sent within two working days for deceased patients over 4.5 months This run chart displays the percentage of discharge summaries (DCS) for deceased patients sent within the target time of two working days from discharge. Intervention at red line. There is a clear, but transient increase in the percentage of summaries sent within the target time after the intervention.

The project’s first intervention, targeting RIP discharge summaries, achieved an increase in primary outcome from 50% pre-intervention to 80% two weeks post-intervention (Figure 4, intervention at red line).

**Figure 5:**
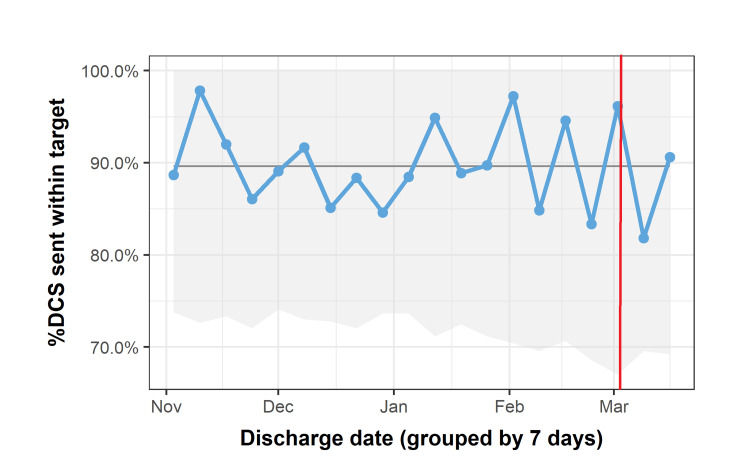
Percentage of discharge summaries sent within two working days for patients discharged from the respiratory ward This run chart displays the percentage of discharge summaries (DCS) for patients from respiratory wards within the target time of two working days from discharge. Intervention at red line. There is no clear response to the intervention.

Unfortunately, this was not sustained, and practice returned to pre-intervention levels within six weeks. The second intervention based on patient lists and targeting the respiratory wards had no effect on the primary outcome measure (Figure 5, intervention at red line). Junior doctors who had implemented this change felt that though they were more aware of patients discharged without summaries sent, their workload was such that they were unable to act on this. They were able to disregard the extra patients on their list by sorting it by bedspace; in this way, discharged patients fell to the bottom and were more easily ignored. Awareness of balancing factors was important, and enacting a change that was perceived to increase workload in the midst of the Coronavirus-19 pandemic led to a perhaps inevitable outcome.

**Figure 6:**
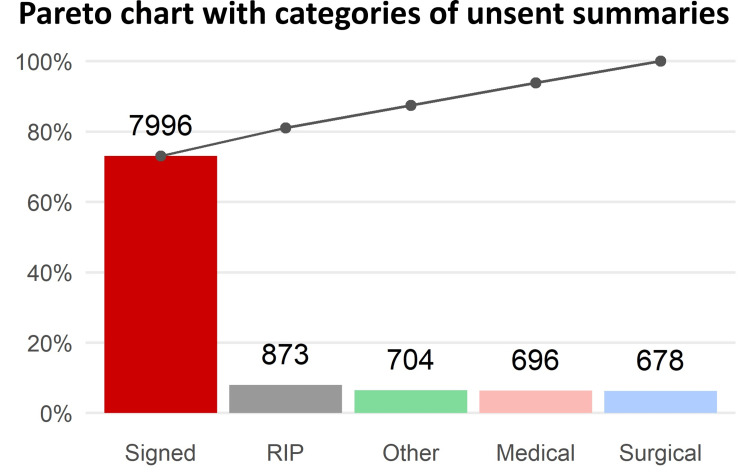
Pareto chart showing relative importance of categories of unsent discharge summaries This Pareto chart shows the impact of change in certain areas. By far the greatest impact would be realised by automatic sending of signed discharge summaries, followed by patients discharged as deceased.

Another potential contextual issue with this intervention was the timing, just before the changeover of junior doctor teams. Shortly after the intervention was implemented, all the junior doctors involved moved to different wards, and the new team may not have been aware of the new list. This would also indicate that the existing cohort did not find it helpful enough to pass on to the incoming team. The third area for improvement, automatic sending of signed discharge summaries, was regrettably not implemented by the end of our quality improvement project period. However, significant progress has been made by collaborating with other hospitals and creating the Pareto chart (Figure 6), which illustrates the substantial benefits such a change could potentially confer.

## Discussion

This project describes a three-pronged approach to addressing the unacceptable delay in sending of hospital discharge summaries by hospital doctors to general practices after discharge. Although respectively successful but not sustainable, not successful and not implemented during the study duration, this project has yielded a number of valuable insights into the issue of unsent discharge summaries, as well as into the quality improvement process more widely.

Firstly, it is clear from the second PDSA cycle that awareness of the issue is not a limiting factor in this case. The list-based intervention was designed to make unsent discharge summaries more visible, based on the assumption that doctors would be willing and able to write and send them if they knew which were missing. The lack of change following this intervention, as well as the subjective feedback received from doctors taking part, revealed that ignorance was a lesser obstacle than perceived addition to an overstretched workload. This would imply that any educational approach would be similarly unhelpful, and instead a successful intervention would make it easier to write and send summaries (or more difficult to fail to do this).

This is further demonstrated in the “RIP summaries” cycles. Educational interventions are known to suffer from unsustainability and the poster-based intervention within the medical examiner’s office was no exception. For an intervention to have a long-lasting and significant effect, it must be more than a simple poster or teaching session. It was hoped that by also empowering the medical examiner’s assistants, who were eager to be involved in this change, a “completion culture” regarding RIP summaries would be created. It became clear that even this was not enough to sustainably increase sending. Doctors are well aware of the need to send RIP discharge summaries within two working days, but are limited by high workloads and time pressures. Increasing awareness therefore has little impact, and the next cycle should focus on simplifying or automating the process.

Another limitation was in the way the data was displayed. Statistical process charts (SPC) are an excellent way of graphing quality improvement data and allow visualisation, and calculation, of the significance of any intervention. On reflection, an SPC may have been a more rigorous way of displaying the project’s data. On the other hand, this project’s main asset has been its method of data collection which has allowed not only the assessment of the scale of the problem but also tracking of responses to interventions in fine detail. Constructing the report to facilitate this required some basic knowledge of programming, but it is now accessible to other investigators and has been highlighted to the Trust team as a powerful tool for further work addressing discharge summary deficits.

The third attempted intervention in this project was the automated sending of signed discharge summaries, a change that required authorisation and programming skill beyond the project team members’ capabilities. This therefore led to much discussion in various different meetings and forums, and ultimately some resistance. Though the Pareto chart demonstrated evidence of its potential efficacy, and senior management displayed strong motivation to improve summary sending, this discussion took place throughout most of the nine months over which this project was run. These obstacles conveyed a valuable lesson; how does one continue working on a problem knowing that the ideal solution is out of reach? This is a frustration many projects must encounter, and require team members to value even ‘small wins’ as per Karl Weick [16], or the possibility of such.

Aside from this third intervention not actually being implemented during the study period, other barriers to its potential success remain. Discharge summaries are often prepared in advance of a patient leaving, especially if they are awaiting social care or transport as opposed to further medical intervention. In practice, this means that the summary is signed, but not sent. The problem with this, as alluded to above, is the lack of continuity in rota patterns; the doctor who prepared the discharge summary may not be at work when the patient is actually discharged. Several colleagues volunteered that if this change were to be enacted, the break in the chain between signing and sending may simply be pushed back earlier and that summaries will not be signed in the first place. The hesitancy around completing the process is due to the fast-changing nature of an acute hospital. For example, the “medically optimised” patient awaiting a package of care may fall over in the bathroom and sustain a fracture or contract a hospital-acquired infection. Sending the summary before the patient physically leaves the hospital risks an out-of-date summary being sent irretrievably to the GP.

To counter this reasonable concern, it was proposed that a “delay” be implemented, whereby all signed discharge summaries will enter a "queue" and automatically be sent at midnight or after 24 hours. An alternative is that they are only released from the queue when the patient has physically left the hospital. In this way, doctors would have time to go back and amend (or cancel) the discharge summary, increasing the likelihood that it is fully accurate when the patient leaves. Talks to implement this change, as part of the third cycle, are still ongoing. It is hoped that collaboration with other hospitals that also use Epic and have already introduced such a system may provide enough confidence and impetus to make this change.

## Conclusions

The importance of the discharge summary is well evidenced, both anecdotally and in literature. This simple document is usually the only handover between secondary and primary care, providing vital information about often complex admissions, but also detailing important instructions and recommendations for follow-up care. Ensuring the discharge summary is sent in a timely manner means that any such instructions can be enacted efficiently; failure to do so is potentially dangerous and could lead to readmission. The project aimed to increase the percentage of discharge summaries sent to GPs within two working days to 90% across all departments by 14th July 2022. During the study period, three separate areas were targeted based on the initial data and root cause analysis. In each, PDSA cycles were designed and enacted with varying success. Areas targeted included not just the worst performing, but those with high numbers of discharge summaries sent and the largest single cause of the deficit as shown by the Pareto chart. The root cause analysis provided a valuable source of inspiration for each intervention. Each cycle benefited from continuous, day-to-day measurement that allowed close monitoring of success or failure.

The project failed in achieving its primary aim. By the end of the measurement period, it was clear that little if any permanent progress had been made in improving outcomes. However, what the project did achieve was collection and stratification of a large volume of useful data. By utilising the as-yet untapped potential of the hospital’s electronic patient record system, a vast gold mine of anonymised data was accessed and displayed as striking graphs and figures. Such figures were shared with members of the trust and key stakeholders, who recognised the significant achievement this represented. Not only does this data pave the way for further PDSA cycles and tests-of-change, but allows continuity for the next group of doctors who share a desire to tackle this problem. Now that the reports have been created, it is a small matter to gather data and analyse it on a regular basis. This should allow future groups to hit the ground running, taking on this issue with a wealth of data already behind them.
